# Self‐Immolative Systems in Diagnostic and Therapeutic Applications

**DOI:** 10.1002/cmdc.202500858

**Published:** 2026-03-16

**Authors:** Windbedema Prisca Ouédraogo, Thomas Cailly, Valérie Collot

**Affiliations:** ^1^ Laboratoire de Développement du Médicament (LADME), CEA‐CFOREM Université Joseph KI ZERBO Ouagadougou Burkina Faso; ^2^ UFR Sciences de la Santé Université Joseph KI ZERBO Ouagadougou Burkina Faso; ^3^ Université Caen Normandie Normandie Univ, CERMN UR4258 Caen France; ^4^ Université Caen Normandie Normandie Univ, CYCERON UAR 3408‐US50, IMOGERE Caen France; ^5^ Institut Blood and Brain @Caen Normandie (BB@C) Caen France; ^6^ Department of Nuclear Medicine CHU Côte de Nacre Caen France

**Keywords:** chemosensors, drug delivery, nanoparticles, polymers, prodrugs, profluorophores, release, self‐immolative

## Abstract

Self‐immolative systems first emerged in prodrug chemistry in the 1980s. Since then, several types of self‐immolative systems have been developed. Despite their structural differences, all self‐immolative systems operate on the same principle: an intramolecular reaction cascade triggered by a specific stimulus, ultimately leading to the release of a molecule of interest. Self‐immolative systems offer the possibility of delivering molecules safely, ensuring their specific, residue‐free release at a defined location. Consequently, they have been applied in various fields, including targeted drug delivery, detection of protein biomarkers and small endogenous molecules, signal amplification, and the engineering of nanomedicines. Self‐immolative systems therefore represent a versatile platform for chemical engineering in biomedical sciences.

## Graphical abstract

Self‐Immolative Systems enable the precise, residue‐free release of active molecules, drugs, probes, or sensors, via cyclization or elimination, triggered by specific stimuli. Their versatility drives advances in drug delivery, diagnostics, nanotechnology, materials chemistry, and biosensing, offering chemists control over molecular activation and shaping the future of targeted therapies and smart materials.

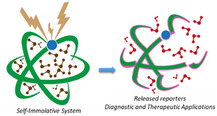

## 1 Introduction

Over the past four decades, the use of self‐immolative systems has increased significantly, and their fields of application have diversified. Widely used in biomedical sciences, self‐immolative systems have been an innovative breakthrough in pharmaceutical and medical research. These systems are known by various names; some refer to them as self‐immolative linkers [1, 2, 3, 4, 5, 6, 7, 8, 9], while others call them self‐immolative spacers (SIS) [10, 11, 12, 13]. Regardless of the terminology the role of these systems remains consistent. The concept of self‐immolative systems was first introduced by Philip and coworkers, who described them as molecular connectors designed to link a carrier moiety to a therapeutic agent. This linkage enhances the overall stability of the final molecule while enabling a controlled and efficient release of the active drug moiety in response to a specific stimulus at the target site [14].

In this work, the term self‐immolative systems refers to both self‐immolative linkers and SIS. Over the years, self‐immolative systems have emerged as a key tool in various scientific domains, including drug delivery (particularly for anticancer agents) [4, 15, 16], bioanalysis and biosensing [16, 17, 18], as well as gated materials engineering and nanoparticles [19, 20, 21, 22]. This review provides an overview of the fundamental principles and diverse applications of self‐immolative systems. Specifically, it addresses the following key questions: What defines a self‐immolative system? How does it work? Furthermore, readers will discover a range of self‐immolative systems applications, offering both inspiration and practical guidance for selecting the most suitable self‐immolative systems in their respective fields.

### 1.1 Self‐Immolative Systems Conception

**SCHEME 1:**
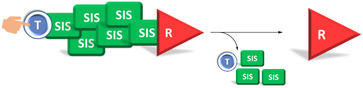
Self‐immolative systems topology and principle (Blue: Trigger, Green: SIS, Red: Reporter, the same color code is applied throughout the manuscript.).

Self‐immolative systems can be defined as a molecular system capable of undergoing self‐disassembly upon exposure to a specific stimulus, leading to the release of a reporter. A self‐immolative system typically consists of three main components: a trigger or protecting group, a SIS and a reporter or leaving group (Scheme 1) [10, 23]. The nature of the stimulus is variable, including chemical triggers (e.g. fluoride ions [24], Zn/AcOH [23], hydrogen peroxide [25, 26, 27], acidic or basic pH), physical stimuli (e.g. heat, UV radiation) [24], biochemical activators [16, 24, 26, 27, 28, 29, 30] (e.g. β‐glucuronidase, β‐galactosidase, Penicillin‐G Amidase, cathepsin B, catalytic antibodies, such as 38C2) [16, 24]. The reporter released can be either an active compound (such as a drug) or a probe.

**SCHEME 2:**
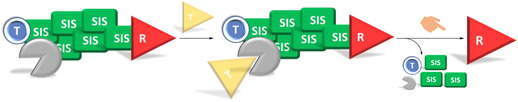
Targeted self‐immolative systems principle (Blue: Trigger, Green: SIS, Red: Reporter, Gray: targeting moiety, Yellow: biological target, the same color code is applied throughout the manuscript).

Researchers have introduced various modifications to this tripartite structure, such as hydrophilic spacers, which will enhance the global solubility or targeting molecules [31, 32]. The latter have found most of their application in the field of oncology, where targeted delivery and improved bioavailability of anticancer agents exclusively within tumor tissues significantly reduce toxicity while enhancing efficiency. In this regard, self‐immolative systems chemistry has enabled the development of a new class of prodrugs by adding a targeting fragment to the typical self‐immolative systems device (Scheme 2). The targeting fragment may be a small molecule such as folic acid or a macromolecule such as shigatoxin B, a receptor ligand, or an antibody. When an antibody is used as the targeting unit, the resulting construct is categorized as an antibody–drug conjugate (ADC) [33, 34, 35, 36, 37, 38, 39].

### 1.2 Self‐Immolative Systems Disassembling

The mechanism of reporter release in self‐immolative systems is similar to that of a firearm. This similarity gives rise to the term ‘trigger’ for the protecting group. Chemically, the release of the reporter occurs due to an increase in entropy and the formation of thermodynamically stable products. Based on the disassembly mechanism, there are two possible pathways for reporter release: elimination and cyclization reactions. When multiple reporters are involved, the release mechanism can involve combination of both elimination and cyclization, multiple elimination, or multiple cyclization reactions.

#### 1.2.1 Elimination Reactions

**SCHEME 3:**
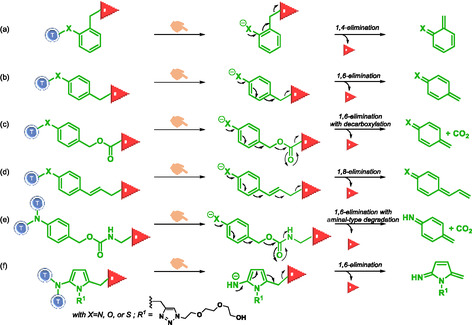
Elimination reactions in self‐immolative systems.

Elimination reactions in self‐immolative systems lead to spontaneous and irreversible disassembly through an “electronic cascade” (Scheme 3). This process occurs via thermodynamically favorable pathway leading to stable products, such as carbon dioxide. In self‐immolative systems, the elimination reaction is designed to release reporter and typically consist of poly‐substituted electron‐rich aromatic species that contain a masked electron‐donor group (e.g. hydroxyl, amine, thiol) positioned at ortho or para position relative to the reporter. The reporter itself, is most often located at the benzylic position. Upon activation, the electron‐donating group generates nucleophilic species. The self‐immolation process then involves cleavage of C—O, C—N, C—S, and C—C bonds. Typical elimination reactions involved 1,4‐elimination (Scheme 3a) [24, 26], 1,6‐elimination (Scheme 3b) [25, 26, 27, 28, 29, 38, 40, 41, 42, 43], 1,6‐elimination with coupled decarboxylation (Scheme 3c) [25, 26, 27, 28, 29, 38, 40, 41, 42, 43], and 1,8‐elimination (Scheme 3d) [16, 26, 44]. Robbins et al. demonstrated that modulation of SIS aromaticity significantly influences release kinetic [45]. Based on these results, Spring et al. and Taddei et al. have more recently designed and developed new SIS that proceed via 1,6‐elimination reactions and enable the release of amides, sulfonamides, thiols, amines, and phenol‐containing molecules [46, 47]. Spring's SIS is based on a *para*‐nitrobenzyl‐amino carbamate scaffold. Reporter release occurs through a 1,6‐elimination followed by decarboxylation and aminal‐type degradation (Scheme 3e). Taddei's SIS is built on a (5‐nitro‐2‐pyrrolyl)methanol scaffold, and reporter release occurs after a 1,6‐elimination (Scheme 3f). Most functional groups involved in 1,6‐elimination type reactions exhibit pKa values around 9, whereas those employed in Taddei's new SIS display higher pKa values.

#### 1.2.2 Intramolecular Cyclization Reactions

**SCHEME 4:**
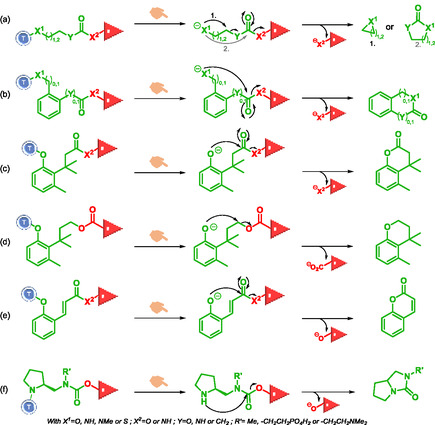
Cyclization reactions in self‐immolative systems.

The structures that promote intramolecular cyclization of self‐immolative systems are most often alkyl chains, pyrrolidine‐carbamate, or bulky aromatic groups substituted in the ortho position (Scheme 4). The alkyl chains contain one or more heteroatoms, such as 4‐aminobutanoyl esters and ethylenediamines. Similar to elimination reactions, intramolecular cyclization occurs through increased entropy and the formation of thermodynamically stable products, such as heterocycles. The cyclization reactions include cyclizations with or without coupled decarboxylation (Scheme 4a) [48, 49, 50]. Cyclization originating from a benzylic group (Scheme 4b) [11, 16, 23, 51], formation 6‐membered cycles from a phenoxide involving either addition‐elimination reactions or nucleophilic substitution reactions (Scheme 4c,d,e) [1, 10, 24]. With pyrrolidine‐carbamate SIS (Scheme 4f), cyclization of the secondary amine triggers carbamate cleavage and reporter release [13, 52, 53]. Dal Corso et al. demonstrated that the latter SIS is the fastest due to the high nucleophilicity of the pyrrolidine nitrogen. The rapid cyclization mechanism leads to the release of cyclic urea and OH‐bearing reporter. Among the factors governing the drug release performance of such amine‐carbamate SIS, the nature of the hydroxyl leaving group is arguably the most important. The higher the degree of substitution of the alcohol (tertiary>secondary>primary), the faster the cyclization [13, 54, 55].

#### 1.2.3 Combined Reactions

In some self‐immolative systems, the disassembling process involves series of elimination or cyclization reactions, and occasionally combination of these reactions. Quinone methide eliminations and cyclization reactions are the two mechanisms most frequently involved. This kind of disassembling has been observed in dendrimers, oligomers, polymers, and gated materials containing a self‐immolative system [24, 43, 48]. The structure of these polymers and dendrimers typically includes a focal point, which is the starting point for disassembling, previously named the trigger. A specific stimulus initiates the self‐disassembly process from ‘head to tail’ through domino‐like chain fragmentation, which involves the release of multiple reporters. Such systems facilitate signal amplification or an increased release of a therapeutic molecule. In some of these polymers, the disassembly upon activation by a specific stimulus is driven by characteristic functional groups such as sulfones, phthalaldehydes, or glyoxylates [24].

## 2 Self‐Immolative Systems in Diagnostics and Therapeutics

### 2.1 From Sensing to Diagnosis

A broad range of biological macromolecules, carbohydrates, lipids, proteins, peptides, and nucleic acids, originating from plants, algae, fungi, animals, or microbial sources, have been investigated as potential biomarkers. Self‐immolative systems have therefore been widely employed for the detection and quantification of biological molecules involved in the diagnosis of various pathologies, including cancers, neurodegenerative disorders, and bacterial infections. In these diagnostic applications, detection is mostly triggered by stimuli originating from small molecules or enzymes, which act as the primary activators of the self‐immolative process [17, 25, 40, 56]. In this section, we illustrate how self‐immolative systems have been evaluated in vitro and, in some cases, in vivo using animal models.

#### 2.1.1 Small Endogenous Molecules as Stimuli

Small endogenous molecules are biologic active molecules with low molecular weight, involved in a wide range of physiological processes. Some of them have been used as biomarkers for diagnostic purposes*.* To date, a variety of small endogenous molecules have been targeted using self‐immolative systems‐based probes, including hydrogen sulfide (H_2_S), hypochlorous acid (HClO), hydrogen peroxide (H_2_O_2_) and reduced glutathione (GSH) [25, 40, 57, 58, 59]. This subsection focuses on several fluorescent self‐immolative probes that have been developed for the detection of key small molecules, with a particular focus on H_2_S, GSH, and H_2_O_2_.

##### 2.1.1.1 Hydrogen Sulfide (H_2_S) Sensing

**SCHEME 5:**
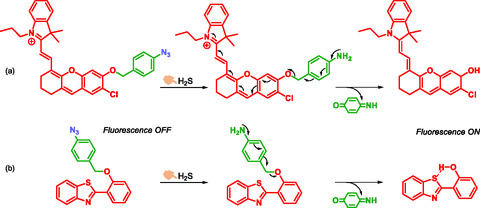
H_2_S detection, using azide trigger and chromophore release by Park (a) and Jiang (b).

Hydrogen sulfide is an endogenous gaseous molecule and a gasotransmitter, similar to nitric oxide (NO) and carbon monoxide (CO). It is recognized as an important biological signaling molecule. Increased levels of H_2_S have been detected in sepsis, while decreased levels have been observed in Alzheimer's disease. Therefore, quantifying H_2_S concentrations could aid disease diagnosis. Park et al. developed a near‐infrared azide‐based probe, NIR‐Az, for detecting H_2_S [40]. This tri‐substituted, hydroxanthene‐derived probe features an aromatic azide group at position 7, which H_2_S reduces to a free amine. The resulting amine triggers a self‐immolative cascade, ultimately releasing a fluorescent compound. In contrast, the initial probe exhibits weak or no fluorescence (Scheme 5a). Sensitivity, selectivity, and kinetic studies demonstrate the probe's potential for effective detection and quantification of H_2_S. In vitro, the probe shows good membrane permeability, and in vivo experiments in rats confirm its fluorescent emission. Based on these results, the probe developed by Park et al. exhibits a promising profile for H_2_S detection in biological samples such as blood and tissue homogenates.

Jiang et al. were among the first to develop a selective fluorescent probe whose incorporating an azide group within the self‐immolative system, specifically designed to respond to hydrogen sulfide. In this probe, fluorescence emission is based on excited state (Excited state intramolecular proton transfer = ESIPT) [41]. ESIPT chromophore is 2‐(2′‐hydroxyphenyl)‐benzothiazole (HBT). In the presence of hydrogen sulfide, the probe undergoes a self‐immolative 1,6‐elimination, releasing the fluorescent HBT chromophore (Scheme 5b). In vitro studies show that the fluorescence intensity increases with the concentration of sodium hydrogen sulfide (NaHS). Cells incubated with the probe alone emit significantly less fluorescence compared to cells exposed to both the probe and NaHS. These results indicate that the probe enables highly sensitive and selective detection of H_2_S.

##### 2.1.1.2 Reduced Glutathione (GSH)

**SCHEME 6:**
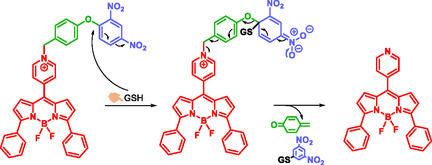
Selective reaction of BODIPY‐based probe to GSH and self‐immolation by Zhang.

Zhang et al. developed a probe based on the BODIPY (Boron‐dipyrromethene) derivative. This probe incorporates a BODIPY fragment linked to a *para*‐dinitrophenoxybenzylpyridinium moiety that functions as SIS (Scheme 6) [29]. The probe selectively detects reduced glutathione which is a tripeptide known as a biomarker of oxidative stress [60]. Pathologies such as AIDS, cancer, liver damage, and neurodegenerative diseases show abnormal levels of reduced glutathione [61]. In the presence of the probe, the reduced glutathione reacts through an aromatic nucleophilic substitution on the dinitrophenyl moiety, triggering a self‐immolative electronic cascade. This leads to the disassembly of the SIS and the subsequent release of the highly fluorescent BODIPY dye [29]. Due to its low cytotoxicity, the probe is well suited to the detection of GSH in living cells. Building on the same principles of BODIPY and self‐immolation, Zhang's group reported in 2018 a probe capable of simultaneously detecting hydrogen sulfide (H_2_S) and organosulfur compounds such as GSH, cysteine, and homocysteine [59]. This multianalyte detection strategy highlights the versatility of the BODIPY/ self‐immolative systems combination for sensing biologically relevant thiols.

##### 2.1.1.3 Hydrogen Peroxide (H_2_O_2_)

**SCHEME 7:**
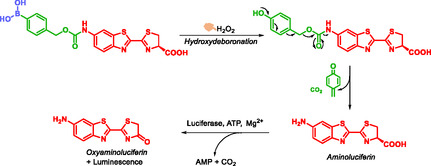
H_2_O_2‐_Induced Aminoluciferin release by Wu.

H_2_O_2_ is a key component of reactive oxygen species (ROS), and is involved in various physiological processes, such as cell signaling, energy metabolism, cell growth, cell metabolism, cell aging. It is also implicated in various pathologies including cancer, cardiovascular disease, Alzheimer's disease and Parkinson's syndrome [25]. Wu et al. synthesized and evaluated, in vitro and in vivo, a bioluminescent probe for the imaging H_2_O_2_ detection. This probe is composed of three parts: an aromatic boronic acid moiety sensitive to H_2_O_2_, SIS and a bioluminescent aminoluciferin fragment (Scheme 7) [25]. The bioluminescence mechanism involves two sequential steps: first, the boronic acid is oxidized by H_2_O_2_; second, the released aminoluciferin is activated by luciferase, leading to light emission. In vitro analyses show that the probe exhibits high sensitivity and selectivity toward H_2_O_2_ compared to other ROS. Sensitivity assays further demonstrate a linear correlation between H_2_O_2_ concentration and the intensity of the emitted luminescence. Both in vitro imaging in live cells and in vivo imaging in tumor‐bearing mouse models confirm that the probe is an excellent candidate for the real‐time detection of H_2_O_2_ in biological systems. However, probes that emit bioluminescence through a single‐step reaction could offer advantages in terms of simplicity and reaction speed.

#### 2.1.2 Enzymes as Stimuli

Because of their high specificity and catalytic efficiency, enzymes constitute particularly attractive biomarkers for diagnostic applications. To date, numerous self‐immolative probes have been designed to respond selectively to enzymatic activity by undergoing a trigger‐induced cleavage followed by a cascade fragmentation, leading to the release of an easily detectable reporter. This approach has been widely implemented in fluorescent probes, where enzyme‐mediated activation produces a strong and quantifiable signal enhancement. In this subsection, we highlight representative examples of self‐immolative probes developed for the detection of key enzymatic biomarkers, with particular attention to β‐galactosidase, ALP, and cytochrome P450 isoforms. These enzymes play central roles in numerous metabolic and regulatory pathways and are often dysregulated in diseases such as cancers, inflammatory disorders, or neurodegenerative conditions.

##### 2.1.2.1 β‐Galactosidase

**SCHEME 8:**
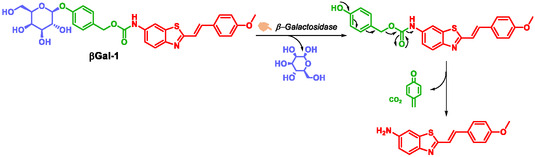
βGal‐1 probe self‐immolation by Goberhan.

β‐Galactosidase is an enzyme that hydrolyzes glycosidic bonds in β‐galactosides(gangliosides and other β‐conjugated galactosides) at the β‐terminal position. Goberdhan et al. demonstrated that β‐galactosidase activity is detectable in senescent cells at pH 6, a phenomenon now known as senescence‐associated β‐galactosidase (SA‐βGal) activity. This activity is widely recognized as a marker of cellular senescence [62, 63, 64]. One of the major obstacles in anticancer chemotherapy is chemoresistance, which may be either intrinsic (preexisting) or acquired (emerging after initial treatment) [65]. This resistance is frequently linked to intratumoral heterogeneity and represents a significant barrier to therapeutic efficacy [65, 66]. Interestingly, many cancer cells retain the capacity to undergo therapy‐induced senescence (TIS), positioning senescence induction as a promising strategy to circumvent chemoresistance and enhance treatment outcomes [67]. In this context, Filho et al. developed profluorophores designed to detect SA‐βGal activity, aiming to establish a platform for high‐throughput screening of senescence‐inducing compounds. Among their library of candidates, one compound, designated βGal‐1, exhibited particularly promising performance [64]. Structurally, βGal‐1 is a tripartite molecule consisting of a β‐*D*‐galactopyranoside moiety linked via its hydroxyl group to the SIS, parahydroxybenzylcarbamate. The fluorophore, (*E*)‐2‐(4‐methoxystyryl)benzo[d]thiazol‐6‐amine is linked to the SIS through an amide bond (Scheme 8). Under simulated physiological conditions (PBS buffer, pH = 7.4) with 1 U/mL of β‐galactosidase during 10 min),enzymatic activation and fluorophore release were induced, *β*Gal‐1 exhibits a fluorescence signal ≈200‐fold higher than βGal‐1 in the same simulated physiological conditions without the enzyme β‐galactosidase. Furthermore, βGal‐1 displays exceptional stability across a broad pH range, retaining structural integrity for up to 96 h. In vitro assays confirmed its high enzymatic specificity, as no other enzymes tested were capable of hydrolyzing βGal‐1. In induced‐senescence cancer models, βGal‐1 enables real‐time, prolonged monitoring of SA‐βGal activity, maintaining signal intensity over several hours. Compared to existing SA‐βGal detection methods, βGal‐1 presents multiple advantages: it requires no pretreatment prior to imaging, is suitable for live‐cell applications, and possesses a low detection threshold.

**FIGURE 1:**
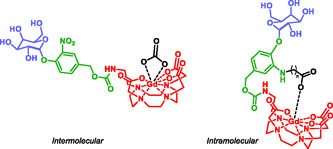
Inter versus intramolecular activation of q‐modulated MRI contrastagents sensitive to β‐galactosidase. In the intermolecular design, endogenous CO_3_^2−^ binding is optimized through manipulation of structural isomers whereas the intramolecular design incorporates a pendant carboxylate ligand with variable linker length to maximize coordination.

Magnetic resonance imaging (MRI) is a powerful clinical and research tool giving excellent spatiotemporal resolution and an unrestricted depth penetration [68, 60]. However, the development of bioresponsive contrast agents capable of detecting enzymatic activity in vivo remains an ongoing challenge. In this context, Lilley et al. designed a novel series of self‐immolative MRI probes for the rapid and specific detection of β‐galactosidase activity in murine models (Figure 1) [69]. These probes are based on two distinct molecular architectures that respond to β‐galactosidase by modulating the coordination dynamics of water molecules around gadolinium(III) ion [Gd(III)], a widely used MRI contrast agent. Upon enzymatic activation, both probe designs trigger an electronic cascade that ultimately releases a common MR‐active species, resulting in a detectable MRI signal. This strategy lays the groundwork for the development of next‐generation bioactivated MRI agents capable of high‐precision imaging of disease‐associated enzymatic activity.

##### 2.1.2.2 Glycosidases

**FIGURE 2:**
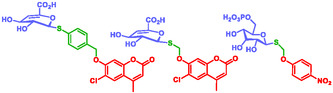
Glycosidase triggered SIS by Nasseri.

Glycosidases are also involved in pathologies such as AIDS, cancer, Alzheimer's disease, and Gauchers’ disease. Nasseri et al. developed fluorogenic thioglycoside substrates featuring thiol‐based self‐immolative linkers for the selective screening of metagenomic libraries of unconventional glycosidases activities (Figure 2) [70].

##### 2.1.2.3 Alkaline Phosphatase (ALP)

**SCHEME 9:**
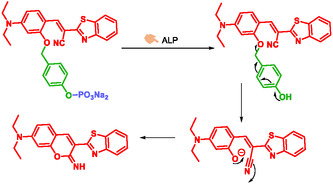
Structure and self‐immolation mechanism of fluorogenic probe for ALP sensing.

Park et al. designed a tripartite fluorogenic probe comprising a fluorescent structure, a SIS, and an alkaline phosphatase sensitive trigger. ALP is an enzyme that catalyzes the hydrolysis of phosphoesters. Its activity is commonly utilized in the diagnosis of specific hepatobiliary and bone pathologies [71]. In this probe, self‐immolative system increases the affinity of the substrate toward ALP [28]. In the presence of the probe, ALP catalyzes the hydrolysis of the PO bond, followed by 1,6‐elimination of SIS and intramolecular cyclization leading to the formation of benzothiazole iminocoumarin, a highly fluorescent molecule (Scheme 9). The probe not only detects ALP but also quantifies its activity. Additionally, it allows for the screening of inhibitors for potential use as therapeutic agents [28]. Given that the phosphate moiety is basic, a potential limitation of this probe could be the occurrence of acid–base reactions in acidic media. Therefore, it is essential to undertake studies on the stability of this phosphate fragment under varying pH conditions.

##### 2.1.2.4 Cytochrome P4502J2

**SCHEME 10:**
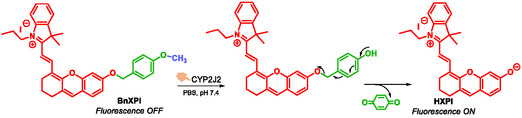
Mechanism of CYP2J2 triggering the fluorescence response of BnXPI.

Cytochromes P450 are hemo‐mono‐oxygenases belonging to a superfamily of liver enzymes that play a crucial role in the oxidative metabolism of various endogenous and exogenous molecules, including drugs. They are divided into families and sub‐families [72, 73]. Among them, CYP2J2 is known to metabolize polyunsaturated fatty acids into epoxyeicosatrienoic acids (EETs), bioactive lipids that promote cell proliferation and angiogenesis. High expression levels of CYP2J2 have been observed in several solid cancers (esophagus, breast, liver, lungs...) and are associated with increased levels of EETs in the body fluids affected patients [74, 75, 76]. Overexpression of CYP2J2 promotes cell proliferation, survival, and migration, while EETs stimulate angiogenesis in primary tumor tissues and support metastatic progression [74, 77, 78, 79]. Consequently, CYP2J2 has emerged as a promising biomarker for cancer. After the synthesis and screening of a series of fluorescent probes, Ning et al. identified BnXPI as the most effective probe for the real‐time detection of CYP2J2 activity [80]. Incorporation of a para‐hydroxybenzyl as SIS into the structure of BnXPI enabled the optimal positioning of the *O*‐methyl group within the CYP2J2 active site, thereby enhancing enzymatic specificity. Upon *O*‐demethylation by CYP2J2, the probe undergoes self‐immolation, leading to the release of the fluorescent molecule (*E*)‐2‐(2‐(6‐hydroxy‐2,3‐dihydro‐*1H*‐xanthen‐4‐yl)vinyl)−3,3‐dimethyl‐1‐propyl‐*3H*‐indol‐1‐ium (HXP), which subsequently emits fluorescence (Scheme 10). In vitro and in vivo sensitivity and selectivity studies confirmed that BnXPI is a highly effective diagnostic tool for detecting cancers that overexpress CYP2J2. Additionally, it represents a valuable tool for investigating the biological role of CYP2J2 in cancer and may support the development of novel therapeutic strategies.

##### 2.1.2.5 Furin Proprotein Convertase

**SCHEME 11:**
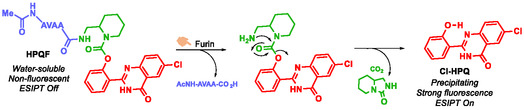
Chemical structure of activatable probe HPQF and self‐immolative mechanism after furin triggering.

Furin is a protease belonging to an ancient family of proprotein convertases of the subtilisin / kexin type (PCSK) [81]. It activates over 150 proproteins of various origins (mammalian, viral, bacterial). Overexpression of this protease has been linked to several cancers (skin, colon, lungs, head, neck, brain…) and is associated with increased tumor aggressiveness, making it a promising cancer biomarker [82, 83, 84]. To enable in situ detection of furin activity, Li et al. developed a fluorescent probe named HPQF [85]. The design of HPQF involves a SIS, piperidin‐2‐ylmethylamine, which links a furin‐specific peptide substrate to the fluorophore 6‐chloro‐2‐(2‐hydroxyphenyl)quinazolin‐4(3*H*)‐one (Cl‐HPQ) (Scheme 11). The SIS confers stability to the probe in the absence of furin and reduces steric hindrance during substrate recognition. In phosphate buffer, the probe remains stable for up to 96 h. Upon enzymatic cleavage by furin, the probe undergoes self‐immolation, resulting in fluorescence emission via an excited‐state intramolecular proton transfer (ESIPT) mechanism, accompanied by fluorophore aggregation. After 4 h of incubation at 37°C in furin‐containing cell lysates, the probe shows a fluorescence increase of up to 62‐fold compared to its initial state, whereas it remains nonfluorescent in the absence of furin.

#### 2.1.3 Various Stimuli

Several profluorophores have been developed for the detection of a wide range of enzymes, including bacterial enzymes and enzymes involved in cancer or skin diseases [30, 64, 86, 87, 88, 89, 90]. In all of these cases, specific recognition of the trigger by the specific enzyme initiates self‐immolation and leads to fluorescence emission. Owing to the remarkable structural versatility of self‐immolative systems, researchers continue to explore and design new SIS motifs to enhance probe performance and enzyme selectivity [91]. In this context, Nakamura et al. developed a novelself‐immolative systems based on a trimethyl carbamate auto‐cleavable linker. Structurally and mechanistically analogous to the trimethyl lock system, this self‐immolative system enables the detection of various enzymatic activities, including those of esterases, ketoreductases, and transaminases.

### 2.2 Drug Delivery Systems

The bioavailability of active compounds is closely related to the efficiency of both the administration route and the drug delivery system. Achieving a desired therapeutic effect depends largely on the bioavailability of the active compounds. In this context, self‐immolative systems chemistry offers two main advantages: the reduction or elimination of off‐target side effects, the obtention of optimal therapeutic outcomes by releasing the active compound specifically at the site of interest. Self‐immolative systems chemistry has been applied across a wide range of therapeutic areas including anti‐cancer, antiviral, antibacterial therapies [35, 38, 39, 58, 92, 93, 94, 95]. In the field of anticancer drug delivery, researchers aim to develop targeted systems that release active agents specifically within tumor tissues. Several strategies have been employed to this end, including controlled‐release systems, ADC, small molecules drug conjugates (SMDC), nanoparticles, liposomes, gels, cyclodextrins, as well as self‐immolative systems releasing the active compound after specific cleavage in the cancerous tissue. Self‐immolative systems ‐based systems are activated by specific cleavage mechanisms in the tumor microenvironment [19, 23, 43, 96, 97, 98, 99, 100, 101].

#### 2.2.1 Self‐Immolating Antibody‐Drug‐Conjugates

**SCHEME 12:**
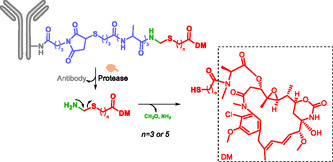
Self‐immolative ADC releasing Maytansinoid.

Monoclonal antibodies have been used as targeting fragment in ADC, to provide mostly anticancer drugs. The antibody targets antigens expressed on the membrane of the cancer cell and/or cells of the tumor microenvironment. In most ADCs, the cytotoxic drug is linked to the antibody via a spacer. In 2025, 17 ADCs (gemtuzumab ozogamicin, brentuximab vedotin, adotrastuzumab emtansine, inotuzumab ozogamicin, polatuzumab vedotin, moxetumomab pasudotox, enfortumab vedotin, trastuzumab deruxtecan, sacituzumab govitecan, belantamab mafodotin, cetuximab sarotolacan, loncastuximab tesirine, disitamab vedotin, tisotumab vedotin, mirvetuximab soravtansine, sacituzumab tirumotecan and datopotamab deruxtecan) have been approved by either FDA (US Food and Drug Administration) or EMA (European Medicine Agency), 13 of them bear cleavable linkers [102]. ADCs enable tumor‐specific delivery by exploiting antibodies that recognize antigens overexpressed on cancer cell membranes and/or cells of the tumor microenvironment [103, 104, 105]. For a more easy and efficient way for intracellular release of the drug, SIS are often integrated into the spacer region [35, 36, 39]. Costoplus et al. developed maytansinoid‐based ADCs bearing peptide‐immolative linkers which enhanced both bystander killing and linker stability in tumor tissues (Scheme 12) [36]. ADCs showed better bystander killing activity and better linker stability, thanks to the self‐immolative system.

**SCHEME 13:**
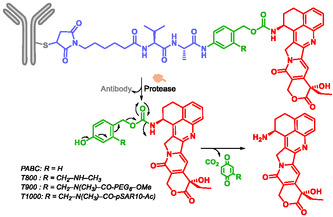
Self‐immolative ADCs releasing Exatecan.

Weng et al. reported ADCs (Scheme 13) combining a self‐immolative moiety and topoisomerase I inhibitor, exatecan, as payload. These conjugates showed potent and sustained activity in both low‐antigen‐expressing and multidrug‐resistant (MDR+) tumors, without increasing toxicity [39]. The self‐immolative moiety without modification, is *p*‐aminobenzyl carbamate (PABC). Modified SIS *p*‐aminobenzyl (pAB), named T moiety by the authors, gave a highly homogeneous and hydrophilic ADC. Depending on the lengh of the T moiety, the resulting analogs were designated T800, T900, and T1000.

#### 2.2.2 Self‐Immolating Prodrugs of Monomethyl Auristatin

**SCHEME 14:**
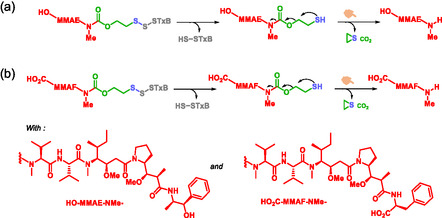
STxB‐drug conjugates of monomethyl auristatin: self‐immolation mechanism of MMAE (a) and of MMAF (b).

To reduce the systemic toxicity of monomethyl auristatin (MMA), several self‐immolative systems‐based prodrugs have been developed [37, 38, 48, 106]. These prodrugs exhibit high stability and effective release upon activation. For example, Batisse et al. designed a targeted delivery system for auristatin derivatives [48]. This system consists of three parts: the nontoxic B subunit of shigatoxin (STxB) as the targeting fragment, SIS which is 2‐mercaptoethyl methylcarbamate, and MMA (E or F) as the active compound (Scheme 14). The receptor for STxB, globotriaosylceramide (Gb3), is overexpressed in several human cancers, including Burkitt's lymphoma and breast, ovarian, testicular, and colon cancers [107, 108]. Two derivatives demonstrated specificity and stability in serum at 37°C. A clinically validated example of self‐immolative MMAE prodrug is Brentuximab vedotin (Adcetris), an ADC that received accelerated approval by the FDA for the treatment of relapsed Hodgkin lymphoma and systemic anaplastic large cell lymphoma (ALCL) [33]. In the European Union, it has also been approved by the EMA for the same indications.

#### 2.2.3 Self‐Immolating β‐Glucuronidase‐Sensitive Prodrugs

**SCHEME 15:**
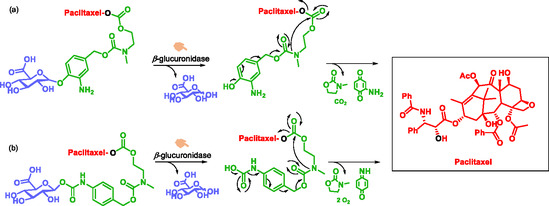
Glucuronide prodrugs of paclitaxel.

Tranoy‐Opalinski et al. reviewed recent advances in the design of β‐glucuronidase‐sensitive prodrugs for selective cancer chemotherapy [43]. Since 1988, Tietze et al. have exploited the tumor‐associated enzymatic activity of β‐glucuronidase to achieve site‐specific drug release [109]. This strategy has yielded a wide range of prodrugs, including derivatives of paclitaxel, histone deacetylase inhibitors, cyclopamine, camptothecin, duocarmycin, MMAE, and anthracyclines. Most rely on SIS derived from para‐hydroxybenzyl alcohol, typically linked via a carbonate, carbamate, or aminoethylcarbamate group [43]. The two most stable paclitaxel prodrugs contain either a masked para‐hydroxybenzyl alcohol (Scheme 15a) or a para‐aminobenzyl alcohol group (Scheme 15b) [110, 111]. These SIS improve prodrug stability, minimize steric hindrance, and enhance enzymatic accessibility, thereby enabling rapid and residue‐free drug release. This work underscores the crucial role of self‐immolative systems in enhancing the targeted bioavailability of anticancer agents.

**SCHEME 16:**
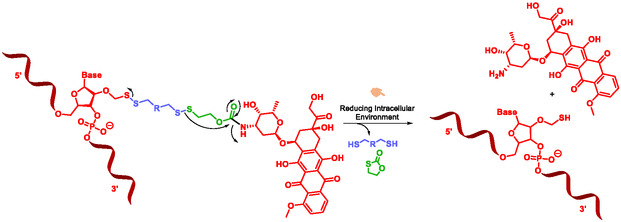
Doxorubicine‐RNA conjugates by Gauthier.

More recently, Gauthier et al. developed a co‐delivery system for chemotherapy, integrating disulfide‐based self‐immolative systems to deliver both small‐molecule drugs and siRNA (Scheme 16). Under the reductive intracellular conditions found in cancer cells, the disulfide‐linked SIS undergoes cleavage, releasing both the drug and the siRNA [8].

#### 2.2.4 Self‐Immolating Small Molecule Drug Conjugates

**FIGURE 3:**
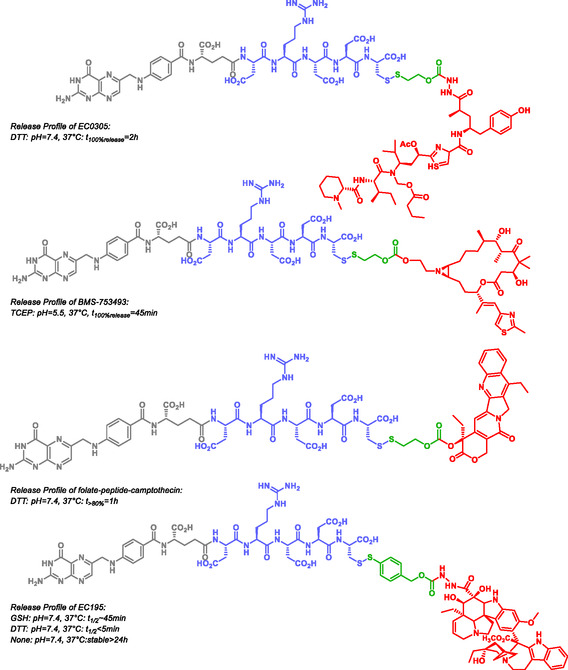
Self‐immolative bioconjugates targeting the folic acid receptor.

Small‐molecule drug conjugates (SMDCs) are similar to ADCs but differ from them by the much smaller size of their homing device, that is, their targeting unit. SMDCs consist of three parts: the homing device, the linker, and the payload, which can be either a drug or a diagnostic agent. The linker can be cleavable or noncleavable and is sometimes modified with a hydrophilicity‐mediating unit. SIS are involved in cleavable linkers, and in SMDCs, para‐aminobenzyl alcohol is the most prominent SIS [112, 113]. Typical homing device for SMDC include glutamic acid urea derivatives targeting prostate specific membrane antigen, folate derivatives targeting folate receptors, aromatic sulfonamides specially targeting carbonic anhydrase IX and peptides targeting integrins, somatostatin receptors, epidermal growth factor receptors, G protein‐coupled receptors, neuropeptide Y receptors or bombesin receptors [112, 113, 114]. All these targets are known to be overexpressed on tumorous cells and or in tumor microenvironments. No SMDS has been approved yet, but many are under preclinical trials or even under clinical trials [113]. Leamon, Walter et al. designed and synthesized a series of several SMDC carrying SIS and targeting the folic acid receptor (Figure 3) [31, 32, 115, 116, 117]. These SMDC consist of folic acid (gray), a hydrophilic peptide chain (blue), the SIS (green) and the active compound (red). Once bound to the folic acid receptor, they undergo internalization via endocytosis. The release tests were performed in phosphate buffer at pH 7.4 and 37°C and in the presence of reducing agents such as L‐glutathione (L‐GSH), (*2S*,*3S*)‐dithiothreitol (DTT), and tris(2‐carboxyethyl)phosphine (TCEP). Experiments at pH 5.5 were performed to mimic the acidic endosomal environment. All conjugates demonstrated efficient drug release upon triggering. During the design and optimization process, the authors also observed improved efficacy when converting the camptothecin to the folate peptide camptothecin prodrug.

**SCHEME 17:**
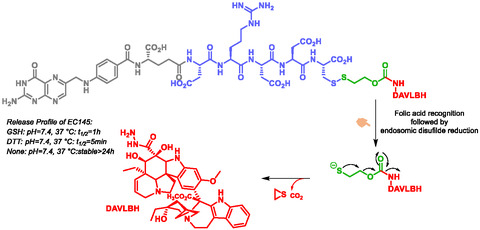
Release profile of EC145 and self‐immolative mechanism.

One of the most avanced examples of self‐immolating SMDC bioconjugates is the EC145, which has progressed through multiple stages of clinical evaluation (Scheme 17) [118, 119, 120]. This compound consists of folic acid conjugated to hydrazine deacetylvinblastine (DAVLBH) via a SIS. Vlahov et al. also reported a pyrrolobenzodiazepine SMDC for cancer therapy [121]. In addition, several other self‐immolative anticancer prodrugs have been described, targeting diverse mechanisms and including conjugates of 5‐fluorouracil, combretastatin, and doxorubicin [92, 122, 123].

**FIGURE 4:**
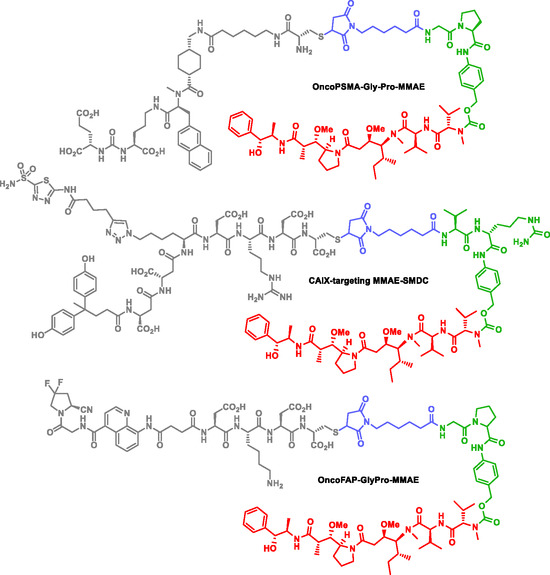
SMDC targeting PSMA, CAIX, FAP.

Cazzamalli et al. have designed and developed various self‐immolating SMDC homing carbonic anhydrase IX, fibroblast activation protein (a stromal tumor‐associated antigen), and prostate specific membrane antigen (PSMA). Para‐aminobenzyl alcohol is the SIS in most of their SMDC. Their most promising SMDC, showing the highest payload release, are those with Gly‐Pro‐para‐aminobenzyl alcohol SIS (Figure 4) [124, 125, 126]. The in vivo efficacy of OncoPSMA‐Gly‐Pro‐monomethyl auristatin E (OncoPSMA‐Gly‐Pro‐MMAE) was evaluated in therapeutic studies conducted either as monotherapy or in combination with an antibody–IL2 fusion protein that preferentially homes to solid tumors. Combination therapy yielded complete and durable responses, underscoring the substantial therapeutic potential of this approach for patients with metastatic prostate cancer.

**FIGURE 5:**
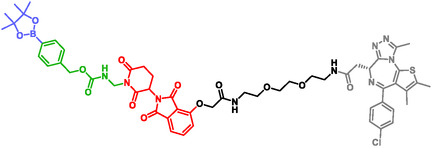
Stimuli‐responsive PROTAC for controlled protein degradation.

Proteolysis Targeting Chimeras (PROTACs) are bifunctional molecules that induce degradation of target proteins by recruiting E3 ubiquitin ligases to the ubiquitin–proteasome system (UPS). Despite their therapeutic potential, systemic administration of PROTACs may cause undesired protein degradation in healthy tissues. To overcome this limitation, An et al. developed stimuli‐responsive PROTACs (sr‐PROTACs) incorporating SIS to enable spatially and temporally controlled activation (Figure 5) [127]. These sr‐PROTACs adopt a modular architecture consisting of a thalidomide‐derived E3 protected by a SIS, a linker, and a target‐binding ligand. Activation can be triggered by exogenous or endogenous stimuli, including UV irradiation, H_2_O_2_, phosphatases, nitroreductases, trans‐cyclooctene, and NADPH:quinone oxidoreductase 1. This strategy enabled selective PROTAC activation in vitro and in vivo, achieving effective degradation specifically within tumor models [127].

#### 2.2.5 Controlled Release: Others Groups of Therapies

Zelikin et al. exploited self‐immolative systemsin the design of anti‐HIV (human immunodeficiency virus) prodrugs. Incorporation of SIS into antiviral prodrugs offers several advantages compared to the parent drugs. These prodrugs exhibit intrinsic antiviral activity prior to the release of the active agent, and their extended half‐life permits reduced dosing frequency, a critical determinant of patient adherence in chronic therapy. The authors applied this strategy to the clinically used antiretrovirals zidovudine and lamivudine [51, 128].

**FIGURE 6:**
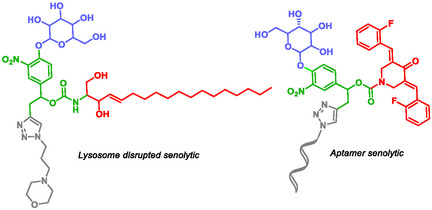
Self‐immolativesenolytics by Xia.

Thiocarbamate‐based self‐immolative systems have also been employed in the development of antibiotic conjugates [129, 130]. Neumann et al. reported a self‐immolative ciprofloxacin conjugate with selective activity against Gram‐negative bacteria [129]. Wang et al. synthesized a self‐immolative conjugate of trimethoprim achieving a molecule with one hundred times more water‐soluble than trimethoprim without SIS [130]. Senotherapy is emerging as a promising approach for the treatment of age‐related diseases and multimorbidity. In this field, senolytic compounds selectively eliminate senescent cells, whereas senostatic agents suppress the pro‐inflammatory signaling associated with senescent cells, which are major contributors to chronic inflammation [131]. Xia et al. designed senolytic prodrugs incorporating SIS to reduce the adverse effects commonly associated with senolytic therapies [132, 133]. The first prodrug (Figure 6, left) comprises a morpholine for lysosome‐targeting unit, a sphingosine as the lysosome‐disrupted drug unit, and a modified β‐galactose as the lysosomal SA‐β‐gal‐responsive connected to the two first units by a SIS. Upon enzymatic activation by SA‐β‐gal in senescent cells (SnCs), the modified β‐galactose unit initiates a self‐immolative cascade, releasing the active drug. The second prodrug (Figure 6, right) incorporates an aptamer targeting the cell adhesion molecule (L1CAM) enabling discrimination between senescent and quiescent endothelial cells. This construct also integrates a SIS, a lysosomal SA‐β‐gal trigger unit and EF24, an inhibitor of antiapoptosis with intrinsic senolytic activity. Both prodrugs demonstrated potent in vitro activity and promising in vivo efficacy in animal models of age‐related diseases.

### 2.3 Theranostics

**FIGURE 7:**
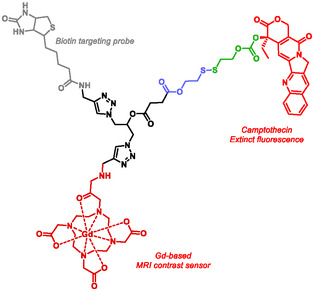
Theranostic by Yang.

The concept of theranostic was introduced by Funkhouser in 2002 to describe the combined use of therapeutic and diagnostic agents. The advantage of this approach lies in its ability to simultaneously visualize diseased tissue, monitor treatment response, and adjust therapy to achieve maximal efficacy and safety, thereby enabling personalized medicine [134, 135, 136]. Several research groups have employed self‐immolative systems for theranostic applications [18, 137, 138, 139, 140]. Yang et al. reported the synthesis of a theranostic prodrug consisting of a targeting agent (biotin), a contrast agent the gadolinium (III) complex (Gd–DOTA), and an anticancer drug (camptothecin) (Figure 7) [139]. These elements were linked via a disulfide‐containing SIS, which undergoes cleavage in the reducing intracellular environment characteristic of cancer cells. Biotin receptors, sodium‐dependent multivitamin transporters, are overexpressed on the surface of many tumors, making them attractive targets for enhancing the efficacy of prodrug strategies. The Gd–DOTA complex, commonly used as an MRI contrast agent, quenched camptothecin fluorescence until drug release occurred through self‐immolation, restoring fluorescence. In the presence of excess of glutathione, the prodrug disintegrated and released the camptothecin, a process confirmed by increased fluorescence. Fluorescence microscopy of HeLa and A549 cells, which overexpress biotin receptors, revealed significantly higher fluorescence compared to normal cells, confirming selective activation.

**FIGURE 8:**
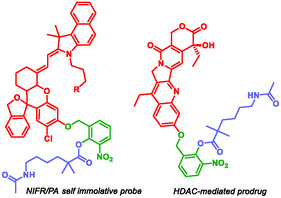
HDAC‐mediated self immolative agents by Liu.

Liu et al. developed a self‐immolative probe and drug those enable in vivo activatable near‐infrared fluorescence (NIRF) and photoacoustic (PA) imaging and drug released triggered by Histone deacetylase (HDAC) [95]. The SIS relied on a finely tuned phenyl ester linker, in which nucleophilicity and steric hindrance were optimized by introducing an ortho electron‐withdrawing substituent and an *α*‐geminal dimethyl group. This SIS was cleverly connected to a NIRF/PA probe designed and developed by the authors (Figure 8). This probe successfully detected HDAC upregulation in tumor cells and identified the efficacy of HDAC inhibitors in vivo. Furthermore, the authors synthesized a prodrug by linking their SIS to SN‐38, the active metabolite of irinotecan, a topoisomerase I inhibitor widely used in the treatment of various carcinomas. This work highlights the potential of self‐immolative systems to provide innovative tools for theranostic.

**FIGURE 9:**
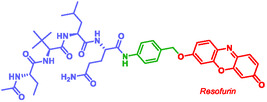
Self‐immolative fluorescent probe by Xu.

Xu et al. reported a self‐immolative fluorescent probe (MP590), designed for the detection and imaging of the SARS‐CoV‐2 main protease M^pro^ [141]. This MP590 consists of three parts: a M^pro^‐cleavable peptide sequence (N‐acetyl‐Abu‐Tle‐Leu‐Gln), a fluorophore (resorufin), and an aromatic SIS (4‐aminobenzyl alcohol PABA) (Figure 9). Upon proteolytic cleavage, the SIS underwent self‐immolation, releasing resorufin and enabling fluorescence. The mechanism was validated by HPLC and mass spectroscopy. MP590 allows both quantitative and qualitative evaluation of M^pro^ activity and inhibition in infected cells [141].

### 2.4 Gated Materials for Controlled Release and Amplified Release

In this section, we aim to describe the process followed by researchers to go from self‐immolative oligomers to smart nanostructures featuring pores, named gates which enable on‐demand release of their contents.

#### 2.4.1 Self‐Immolating Polymers (SIPs)

SIPs were first designed and synthesized by Shabat et al. [142]. These SIPs sequentially disassemble into their building blocks once the process is initiated. SIPs are the culmination of work by the groups of Shabat et al. [143], Sheeren [144], McGrath [145] going from oligomers, dendrimers to self‐immolative comb‐polymers [146]. Subsequent innovations have led to the design and development of hybrid systems in which SIPs are conjugated to nonSIPs, nanoparticles, or antibodies, enabling applications in diagnostic and drug delivery. SIPs can thus be broadly classified into oligomers, dendrimers, and linear or true polymers. Initially conceived for targeted drug delivery via prodrugs ensuring controlled release of active agents, SIPs have since been widely applied to amplify molecular release signals. Oligomers contain two to three SIS undergoing 1,6‐ or 1,4‐elimination and/or cyclization [1, 16, 23, 24].

The appearance of branching in the structure of oligomers marks the transition from self‐immolative oligomers to self‐immolative dendrimers. Dendrimers adopt branched ‘ABn/AnB’ architectures, in which A serves as the initiation point of self‐immolation and B represents molecules of interest [1, 16, 23, 24, 27, 101, 147, 148]. To overcome certain challenges associated with self‐immolative dendrimers, particularly the complexity of multistep synthesis, limited solubility, and steric hindrance, and to avoid an indefinite increase in the number of functional units, linear or ‘true’ SIP have been developed.

**SCHEME 18:**
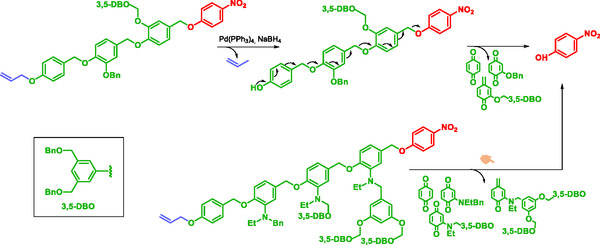
Self‐immolating oligomers by McGrath.

True SIP allow facile one‐step synthesis, improved solubility, and higher payload capacity [24, 149]. SIP have been applied in diagnostic [147, 148, 150], therapy [144, 151, 152], and theranostic [24, 153, 154]. These systems contained three or four para‐hydroxybenzyl alcohol units linked by ether bonds with benzyl ether or benzyl amine substituents at the ortho position (Scheme 18) [153, 154]. Self‐immolation proceeds via 1,6‐elimination mechanism upon trigger removal in the presence of palladium. The reporter molecule, *p*‐nitrophenol, exhibited a characteristic UV signal confirming the self‐immolation process.

**FIGURE 10:**
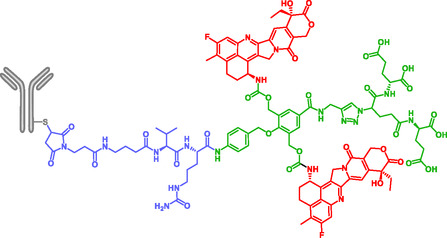
AB_2_ self‐immolative dendrimer by Liubomirski.

More recently, in 2024, Liubomirski et al. developed a novel and highly effective trastuzumab‐based HER2‐targeting ADC using a new dimeric prodrug system based on an AB2 self‐immolative dendritic scaffold (Figure 10) [35]. In this system, exatecan is the chemotherapeutic drug, while a Val‐Cit dipeptide acts as the cathepsin B‐cleavage trigger, and a maleimide moiety was introduced at the focal site for antibody conjugation. To reduce hydrophobicity, a tetracarboxylate group was introduced, resulting in a highly effective next‐generation ADC.

**SCHEME 19:**
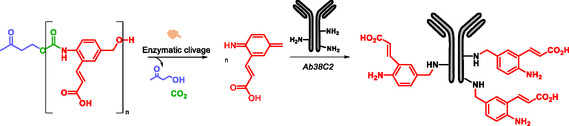
Self‐immolative polymer for protein labeling.

Self‐immolative polycarbamate polymers were first synthesized by Shabat et al. who demonstrated their potential for protein labeling. As proof of concept, the polymers were applied to Penicillin G Amidase (PGA) and the catalytic antibody 38C2 (Scheme 19) [150]. The enzymatic activity of the proteins constitutes the stimulus for the activation of the trigger. This strategy highlights the potential of SIPs to label proteins for diagnostic purpose.

#### 2.4.2 Nanoparticles

**SCHEME 20:**
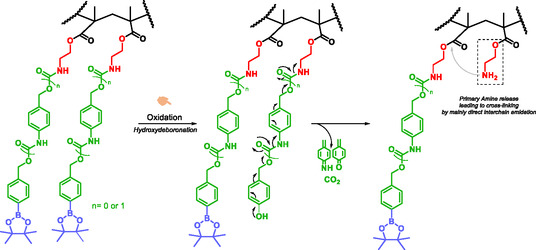
Self‐immolative nanocarriers by Deng.

Nanoscience is a transdisciplinary field that integrates chemistry, physics, biology, medicine… Nanotechnology encompasses the techniques and tools used to manipulate, control, and assemble atoms and molecules into nanoscale materials. The application of nanoscience in “modern medicine” is called “nanomedicine”. Nanotools (such as nanovectors, nanoparticles, nanocarriers) are usually used to ensure targeted delivery and release of active compounds for diagnosis, therapeutic, or monitoring purposes [5, 20, 22, 98, 140, 155, 156, 157, 158]. SIS have been used for the engineering of nanotools. Among the wide variety of nanoparticles available, mesoporous silica nanoparticles (MSN) and gold nanoparticles are the most commonly used. Indeed, they offer the possibility of multifunctionalization of their outer surface. In addition, for MSNs, the silica network provides a large volume allowing the loading of molecules of interest [155, 159]. Deng et al. developed nanovehicles and nanoreactors with a trigger sensitive to hydrogen peroxide and a SIS consisting of 4‐aminobenzyl and 4‐hydroxybenzyl alcohol carbamate for theranostic and diagnostic aim (Scheme 20) [160]. These systems exhibited remarkable stability in the absence of the hydrogen peroxide trigger. For theranostic applications, hydrophobic paclitaxel and hydrophilic doxorubicin hydrochloride were used as active compounds; a MRI contrast agent enabled noninvasive monitoring of the release of active compounds release via a quantifiable signal, was used. For diagnostic purposes, the authors produced several nanoreactors‐based tests incorporating molecules such as 7‐hydroxycoumarin or two dyes, DAPI (Di Aminido Phenyl Indol) of low molecular weight and Texas‐Red‐Dextran (TR‐dextran), or Carboxymethyl dextran (CM‐dextran). These results highlight the significant advantages of self‐immolative systems in nanoscience [160].

**SCHEME 21:**
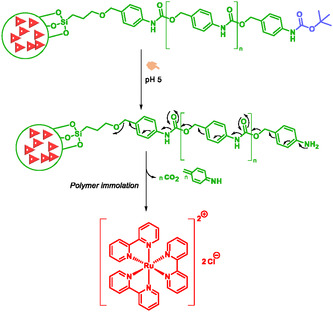
MSN, SIS polymer, and acid‐labile trigger.

Based on the work of Juarez et al. [5], Gisbert‐Garzaran et al. developed MSN [98]. Unlike the first one, the second one puts a 4‐aminobenzyl carbamate polymer as an acid‐labile SIS on the nanoparticles surface (Scheme 21). They loaded their nanoparticles with tris‐(2,2^′^bipyridine)‐ruthenium(II) chloride. At neutral pH and at 37°C, the nanoparticles remained stable and retained their content, while under acidic conditions rapid release was observed due to the self‐immolative polymer. Flow cytometry and fluorescence microscopy analyses confirmed nanoparticles internalization by tumor cells and biocompatibility was demonstrated [98].

**FIGURE 11:**
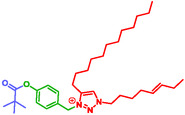
Self‐immolative triazolium‐based amphiphile.

Liu et al. developed a technique for the esterase‐ and protease‐catalyzed self‐assembly and disassembly of gold nanoparticles [22]. This approach holds promise for enzyme. Hollstein et al. designed triazolium‐based amphiphiles, bearing a SIS, capable of siRNA binding, and self‐assembly into nanocarriers (Figure 11) [161]. Upon esterase triggering, the SIS mediated the release of the nucleic acid via self‐immolation.

**FIGURE 12:**
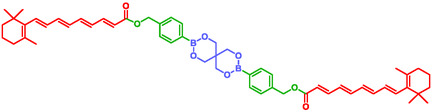
Self‐immolative boronated all‐trans Retinoic acid dimeric prodrug.

Jun et al. designed and synthesized nanomedicine with antithrombotic activity [162]. Self‐immolative systems have been used by these authors in such an elegant way to transform all‐trans Retinoic Acid (atRA) to antithrombotic nanoparticles. They combine two atRA molecules through a boronate SIS (Figure 12). This self‐immolative system is triggered by H_2_O_2_ to release anti‐inflammatory hydroxybenzyl alcohol (HBA), followed by self‐assembly into stable nanoparticles. This innovative use of self‐immolative systems underscores its versatility in nanomedicine.

## 3 Conclusion and Perspectives

Over the past few decades, self‐immolative systems have evolved from simple linkers undergoing elimination or cyclization reactions into sophisticated chemical architectures, including oligomers, dendrimers, and SIPs. These advances have significantly broadened their applications, ranging from residue‐free prodrug activation to the engineering of self‐immolating ADCs, self‐immolating SMDCs, responsive nanomaterials, diagnostic probes, and signal amplification strategies. In particular, SIPs represent a promising frontier in nanoscience, offering opportunities for the controlled self‐disassembly of nanoparticles. In recent years, the design of self‐immolative systems has expanded beyond biomedical contexts to fields such as environmental and electronic sciences [163, 164, 165]. The continuous rise in publications reflects not only a growing scientific interest but also the recognition of self‐immolative systems as essential building blocks for innovative chemical and biomedical engineering. From this perspective, self‐immolative systems can rightly be considered as a cornerstone technology, offering chemists a unique capacity to design systems that combine mechanistic elegancy with practical impact. In the therapeutic and diagnostic fields, several challenges still limit the translational potential of self‐immolative systems. A major hurdle is the validation gap between buffered models and biologically relevant media: predictions made in vitro are often only partially confirmed in plasma, and many self‐immolative systems, although stable under physiological conditions in vitro, show reduced stability in vivo, leading to premature metabolite release and incomplete conversion to the parent drug. Achieving the optimal balance between fast and efficient self‐immolation and sufficient stability during storage and circulation remains a central design challenge. Further progress will require a deeper understanding of selective activation mechanisms, solubility constraints, and pharmacokinetic behavior. Equally crucial is the systematic evaluation of the biological effects of the fragments generated along the self‐immolative cascade, which remain insufficiently characterized despite their importance for safety assessment. Successfully advancing these systems toward clinical translation will depend on addressing the above challenges through integrated chemical, biological, and pharmacokinetic investigations, ensuring that release behaviors observed in controlled environments reliably translate to complex biological settings. The full potential of self‐immolative systems remains to be explored, and continued investigation of their chemical diversity and translational applications is likely to establish them as indispensable tools across medicine, nanotechnology, and beyond.

## Conflicts of Interest

The authors declare no conflicts of interest.
